# The Prevalence of Metabolic Syndrome and Hypertriglyceridemic Waist Based on Sociodemographic Variables and Healthy Habits in Healthcare Workers: A Retrospective Study

**DOI:** 10.3390/life15010081

**Published:** 2025-01-10

**Authors:** Pedro Javier Tárraga Marcos, Ángel Arturo López-González, Emilio Martínez-Almoyna Rifá, Hernán Paublini Oliveira, Cristina Martorell Sánchez, Pedro Juan Tárraga López, José Ignacio Ramírez-Manent

**Affiliations:** 1 Sant Joan University Hospital, 03550 Sant Joan d’Alacant, Alicante, Spain; pedrojav2003@gmail.com; 2 ADEMA-Health Group, University Institute of Health Sciences (IUNICS), 07009 Palma, Balearic Islands, Spain; angarturo@gmail.com (Á.A.L.-G.); emilio@udemax.com (E.M.-A.R.); h.paublini@eue.edu.es (H.P.O.); c.martorell@eua.edu.es (C.M.S.); joseignacio.ramirez@ibsalut.es (J.I.R.-M.); 3 Faculty of Odontology, University School ADEMA-UIB, 07009 Palma, Balearic Islands, Spain; 4 Health Service of the Balearic Islands, 07010 Palma, Balearic Islands, Spain; 5 Faculty of Medicine, Castilla la Mancha University, 02008 Albacete, Castilla-La Mancha, Spain; 6 Faculty of Medicine, Balearic Islands University, 07010 Palma, Balearic Islands, Spain

**Keywords:** metabolic syndrome, hypertriglyceridemic waist phenotype, Mediterranean diet, healthcare worker, physical activity, tobacco consumption

## Abstract

**Introduction**: Metabolic syndrome (MetS) and hypertriglyceridemic waist (HTW) are two multifactorial pathological conditions that have been increasing in prevalence worldwide. The objective of this study was to evaluate how various sociodemographic variables and healthy habits are associated with the presence or absence of MetS and HTW. **Methodology**: This study employed a mixed-methods approach, consisting of a retrospective longitudinal study and a cross-sectional descriptive study, analyzing 44,939 healthcare workers with MS and HTW across four professional categories to evaluate the relationship between age, sex, smoking, physical activity, and adherence to the Mediterranean diet using three diagnostic criteria. Descriptive analysis included categorical and quantitative variables, which were assessed through frequencies, Student’s *t*-test, chi-square, and binary logistic regression models. Logistic regression and Cohen’s kappa were used to evaluate associations and concordances. Age, sex, and lack of physical activity showed the strongest associations with MetS (OR: 2.65–2.84). The results highlight the importance of physical activity and other factors in metabolic prevention. **Results**: Age, sex, and physical activity were the variables most strongly associated with MetS and HTW across the three evaluated diagnostic criteria. The odds ratios revealed significant values: age (9.07–13.71 for MetS and 13.42 for HTW), sex (2.82–3.31 for MetS and 3.72 for HTW), and physical activity (2.65–2.84 for MetS and 2.40 for HTW). **Conclusions**: The risk of developing MetS and HTW among healthcare personnel is influenced by lifestyle habits, sex, and age, with the highest ORs observed in nursing assistants and orderlies. Future research that delves deeper into the causal relationship between lifestyle factors and the severity of MetS and HTW in healthcare personnel will improve understanding and facilitate the development of preventive activities to reduce their incidence.

## 1. Introduction

Metabolic syndrome (MetS) represents one of the primary global public health challenges due to its high prevalence and direct association with cardiovascular diseases (CVDs) and type 2 diabetes mellitus (T2DM) [1]. This cluster of metabolic abnormalities includes insulin resistance, central obesity, atherogenic dyslipidemia, hypertension, and a pro-inflammatory and pro-coagulant state, which synergistically interact to increase the risk of cardiovascular and metabolic complications [2]. Within this context, the hypertriglyceridemic waist (HTW) has emerged as a simple and accessible clinical marker to identify individuals at higher metabolic risk, facilitating the early detection and implementation of preventive strategies [3].

The concept of HTW arises as a screening tool based on the combination of two parameters: waist circumference, which reflects the accumulation of visceral abdominal fat, and elevated plasma triglycerides, a key indicator of atherogenic dyslipidemia [4]. This condition, widely studied in recent decades, is associated with insulin resistance [5], ectopic lipid deposition, and generalized metabolic dysfunction [6]. Its simplicity and correlation with adverse clinical events have driven its adoption in epidemiological research and clinical practice, establishing it as a relevant marker of cardiometabolic risk [7,8].

Over the years, MetS has been the subject of numerous definitions, reflecting the different approaches used to understand its pathophysiology and clinical impact. Early descriptions date back to the 1980s, when Reaven introduced the term “Syndrome X” to describe a cluster of metabolic abnormalities linked to insulin resistance [9]. Subsequently, organizations such as the World Health Organization (WHO), the National Cholesterol Education Program (NCEP-ATP III), and the International Diabetes Federation (IDF) proposed specific diagnostic criteria. While these definitions differ in details, they all include essential components such as central obesity, dyslipidemia, hypertension, and glucose homeostasis disturbances [10].

HTW, on the other hand, is defined as the coexistence of increased waist circumference (indicative of abdominal obesity) and plasma triglyceride levels ≥150 mg/dL [11]. This combination effectively captures two fundamental characteristics of MS: excess visceral adipose tissue and atherogenic dyslipidemia, enabling the identification of at-risk individuals without the need for complex diagnostic methods [12].

The prevalence of MetS and HTW has increased significantly in recent decades, driven by changes in dietary patterns, increased physical inactivity, and population aging [13]. Global estimates suggest that MetS affects a large proportion of adults worldwide, though these figures vary considerably depending on the population studied and the diagnostic criteria used [12]. In Latin America, MetS prevalence is particularly high, exceeding 30% in some countries, a phenomenon linked to a high incidence of abdominal obesity and sedentary lifestyles [14]. Similarly, HTW is present in a substantial percentage of the population, with prevalence ranging from 15% to 40%, depending on factors such as age, sex, and socioeconomic characteristics [5,15].

Epidemiological studies have shown that both MetS and HTW are closely related to the development of CVDs and T2DM [16]. Additionally, HTW has demonstrated significant predictive value for detecting non-alcoholic fatty liver disease (NAFLD) [17], polycystic ovary syndrome (PCOS) [18], and endothelial dysfunction [19], highlighting its clinical relevance beyond cardiovascular risk.

Patients with MetS and HTW often exhibit a series of common clinical features that reflect the interplay of underlying metabolic disturbances. Among the most notable manifestations are abdominal obesity, evidenced by an increased waist circumference, and atherogenic dyslipidemia, characterized by elevated triglyceride and low-density lipoprotein (LDL) levels, along with reduced high-density lipoprotein (HDL) concentrations [12].

From a clinical perspective, central obesity plays a pivotal role, as visceral adipose tissue not only acts as an energy reservoir but also functions as an active endocrine organ that secretes adipokines and pro-inflammatory cytokines. These molecules contribute to insulin resistance, oxidative stress, and systemic inflammation, promoting the development of CVDs and T2DM [20].

Elevated plasma triglycerides, in turn, are associated with the accumulation of triglyceride-rich lipid particles and chylomicron remnants, which are highly atherogenic. The resulting atherogenic dyslipidemia amplifies the risk of atheroma plaque formation and adverse cardiovascular events [21].

It is important to note that both MetS and HTW are associated with a range of comorbidities, including hypertension [22], fasting glucose abnormalities, glucose intolerance [23], NAFLD [24], and endothelial dysfunction [25].

The diagnosis of metabolic syndrome and hypertriglyceridemic waist is based on the identification of key clinical and biochemical parameters. MetS diagnosis relies on criteria proposed by various international organizations, which include the presence of abdominal obesity, dyslipidemia, hyperglycemia, and elevated blood pressure levels.

For hypertriglyceridemic waist, the diagnostic process is simpler, relying solely on the combination of two parameters: increased waist circumference and elevated triglycerides [26]. This simplicity not only facilitates its use in clinical and epidemiological settings but also enables cost-effective and early identification of at-risk individuals.

However, it is important to emphasize that, while HTW and MetS diagnosis can be relatively straightforward, the interpretation of these findings should be conducted within the context of a comprehensive clinical assessment. This includes evaluating additional risk factors, family history of CVDs and T2DM, and the presence of comorbidities.

Among these additional risk factors are the occupational stressors faced by healthcare workers. In the case of healthcare personnel, the constant public exposure of their work, professional relationships with patients, coping with others’ pain, suffering, and death, the need for continuous knowledge updates, the use of new technologies, the current budget cuts in the public healthcare system, staff shortages, and shift work represent occupational stressors that can negatively impact lifestyle habits, increasing obesity, HTW, MetS, and consequently cardiometabolic risk [27,28].

This study highlights the need to recognize and understand certain non-communicable diseases that affect healthcare workers. This group, often idealized for their professional role, faces challenges similar to those of the general population, compounded by stressors inherent to their work environment, as previously detailed. It is crucial to understand how these factors interact with socioeconomic status and lifestyle habits in influencing the risk of developing MetS and HTW.

### Objective

The aim of this study was to evaluate how different sociodemographic variables (age, sex, and socioeconomic status) and healthy habits (smoking, physical activity, and adherence to a Mediterranean diet) are associated with the presence of MetS and HTW in healthcare workers.

## 2. Methods

### 2.1. Participants

This study employed a mixed-methods approach, comprising a retrospective longitudinal study and a cross-sectional descriptive study. A total of 44,939 healthcare workers from various regions of Spain participated. The sample included 14,305 men (31.8%) and 30,634 women (68.2%). Participants were selected from individuals undergoing mandatory annual medical check-ups provided by their employers during the study period. The longitudinal study covered the time frame from 2010 to 2019.

**Figure 1:**
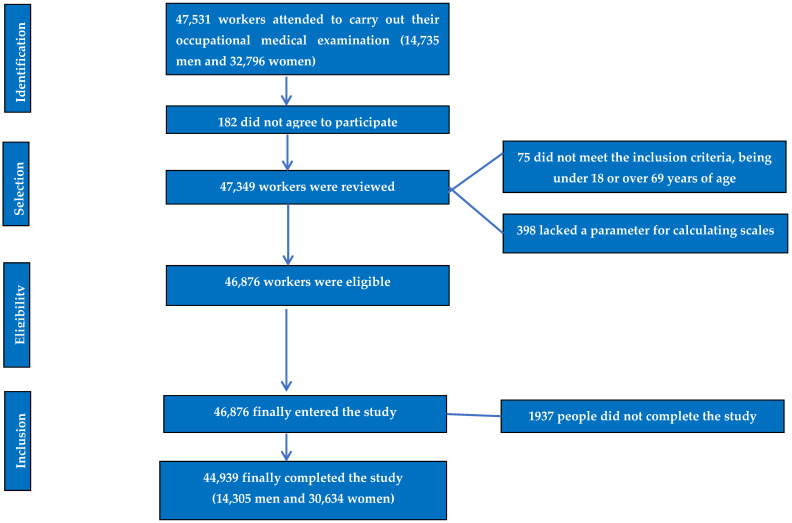
The flow chart detailing the selection and inclusion process for study participants.

Refer to the flow chart in Figure 1 for details.

Inclusion Criteria:

- Aged between 18 and 69 years.
- Employment with one of the participating companies.
- Provided informed consent to participate in the study.
- Authorized the use of their data for epidemiological purposes.

Exclusion Criteria:

- Age under 18 years or over 69 years.
- No employment contract with a participating company.
- Did not provide informed consent to participate in the study.
- Did not authorize the use of their data for epidemiological purposes.

The flow chart of the study participants is presented in Figure 1.

### 2.2. Determination of Variables

Data collection was carried out by occupational health teams from the collaborating companies using the following methodologies:

- Medical History: sociodemographic information (age, sex, occupation) and health-related data such as smoking status, physical activity levels, adherence to the Mediterranean diet, and stress levels were collected.
- Physical and Clinical Measurements: parameters such as height, weight, waist circumference, hip circumference, and blood pressure (systolic and diastolic) were recorded.
- Laboratory Tests: biochemical variables, including lipid and liver profiles and fasting blood glucose levels, were analyzed.

#### 2.2.1. Anthropometric Determinations

To minimize bias, all measurements adhered to standardized protocols. Height and weight were measured using a SECA 700 scale and a SECA 220 stadiometer (SECA, Chino, CA, USA), with participants wearing underwear only. Measurements were taken with the individual in underwear, following international standards for anthropometric evaluation as outlined by ISAK [29]. Data were recorded in centimeters and kilograms.

Waist circumference was measured using a SECA measuring tape, positioned between the lowest rib and the iliac crest. Hip circumference was measured at the widest point of the buttocks with participants relaxed and standing upright [30].

#### 2.2.2. Clinical Determinations

Blood Pressure: It was measured using an OMRON-M3 sphygmomanometer (OMRON, Osaka, Japan) after 10 min of rest in a seated position, ensuring that participants had not consumed food, beverages, or tobacco in the previous hour. Three measurements were taken at one-minute intervals, and the average was calculated.

#### 2.2.3. Analytical Determinations

Blood samples were obtained via venipuncture after a 12 h fast, refrigerated, and processed in reference laboratories within 72 h. Analyses included the following: the determination of triglycerides, total cholesterol, and glucose using enzymatic methods; HDL cholesterol measurement by precipitation; and LDL cholesterol calculation using the Friedewald formula, provided triglycerides were below 400 mg/dL.

#### 2.2.4. Diagnostic Criteria for Metabolic Syndrome

The diagnosis of metabolic syndrome was performed using three different diagnostic models, which were analyzed and evaluated comprehensively:

1. NCEP ATP III (National Cholesterol Education Program Adult Treatment Panel III): MetS is diagnosed when at least three of the following criteria are met:
  ○ Waist circumference > 102 cm in men or >88 cm in women.
  ○ Triglycerides ≥ 150 mg/dL or specific treatment for hypertriglyceridemia.
  ○ Blood pressure ≥ 130/85 mmHg.
  ○ HDL cholesterol < 40 mg/dL in men or <50 mg/dL in women, or specific treatment.
  ○ Fasting glucose > 100 mg/dL or specific treatment for hyperglycemia [31].
2. International Diabetes Federation (IDF): It requires the diagnosis of central obesity, defined as a waist circumference > 80 cm in women and >94 cm in men, along with at least two of the following factors:
  ○ Triglycerides ≥ 150 mg/dL or specific treatment for hypertriglyceridemia.
  ○ Systolic blood pressure ≥ 130 mmHg or diastolic blood pressure ≥ 85 mmHg, or previously diagnosed hypertension under treatment.
  ○ HDL cholesterol < 40 mg/dL in men or <50 mg/dL in women, or specific treatment for this lipid abnormality.
  ○ Fasting glucose > 100 mg/dL or previously diagnosed type 2 diabetes [32].
3. JIS (Joint Interim Statement) defines metabolic syndrome as the presence of at least three of the following risk factors:
  ○ Waist circumference > 94 cm in men and >80 cm in women.
  ○ Triglycerides ≥ 150 mg/dL or specific treatment for hypertriglyceridemia.
  ○ Systolic blood pressure ≥ 130 mmHg or diastolic blood pressure ≥ 85 mmHg, or previously diagnosed hypertension under treatment.
  ○ HDL cholesterol < 40 mg/dL in men or <50 mg/dL in women, or specific treatment for this lipid abnormality.
  ○ Fasting glucose > 100 mg/dL or previously diagnosed type 2 diabetes [33].

Diagnostic Criteria for Hypertriglyceridemic Waist (HTW):

HTW is defined as a waist circumference > 102 cm in men or >88 cm in women, combined with triglycerides ≥ 150 mg/dL or treatment for hypertriglyceridemia.

Operational Definitions

- Professional Categories: Healthcare workers were classified into four groups: physicians, nurses, health technicians (laboratory, pathology, and radiology), and nursing assistants and orderlies [34]. These four groups are representative of the healthcare personnel categories in Spain, and we consider the sample from each group to be nationally representative of their respective category.
- Smoking: It is defined as consuming at least one cigarette per day in the previous 30 days or having quit smoking within the preceding year.
- Adherence to the Mediterranean diet was assessed using the PREDIMED questionnaire [35], which consists of 14 questions, each scored with a value of 0 or 1. A total score of nine or higher indicates high adherence to the Mediterranean diet [36].
- Physical activity levels and sedentary behavior were assessed using the International Physical Activity Questionnaire (IPAQ), which has been validated in over 12 countries, including among Spanish university populations. The questionnaire included seven questions addressing three intensity levels of physical activity (high, moderate, and low) and sedentary behavior. The final result provides the sum of duration (in minutes) and frequency (in days) for low-, moderate-, and high-intensity physical activity, total physical activity, and sedentary time. This self-administered survey evaluates the amount of exercise performed during the past seven days [37].

### 2.3. Statistical Analysis

A descriptive analysis of categorical variables was performed using frequencies and distributions. The Kolmogorov–Smirnov test assessed the normality of quantitative variables, followed by the calculation of means and standard deviations. For bivariate analysis, Student’s *t*-test was used to compare means, while the chi-square test assessed proportions. Variables associated with MetS and HTW were analyzed using a binary logistic regression model, with model fit evaluated using the Hosmer–Lemeshow test. A stratified analysis identified potential confounding factors, but no variables showed significant confounding effects. Cohen’s kappa coefficient assessed concordance between scales. Statistical processing was performed using SPSS version 29.0, with a significance level of 0.05.

## 3. Results

The anthropometric, clinical, analytical, sociodemographic, and healthy habit data of the 44,939 workers included in the study are presented in Table 1. The participants’ average age was slightly above 41 years. Across all variables, lower values were observed in the female group. The population’s mean age ranged between 30 and 49 years. Adherence to a Mediterranean diet was reported by 45.8% of men and 37.9% of women, while 47.5% of men and 38.9% of women engaged in regular physical activity. Smoking was reported by 16.1% of men and 15% of women.

**Figure 2:**
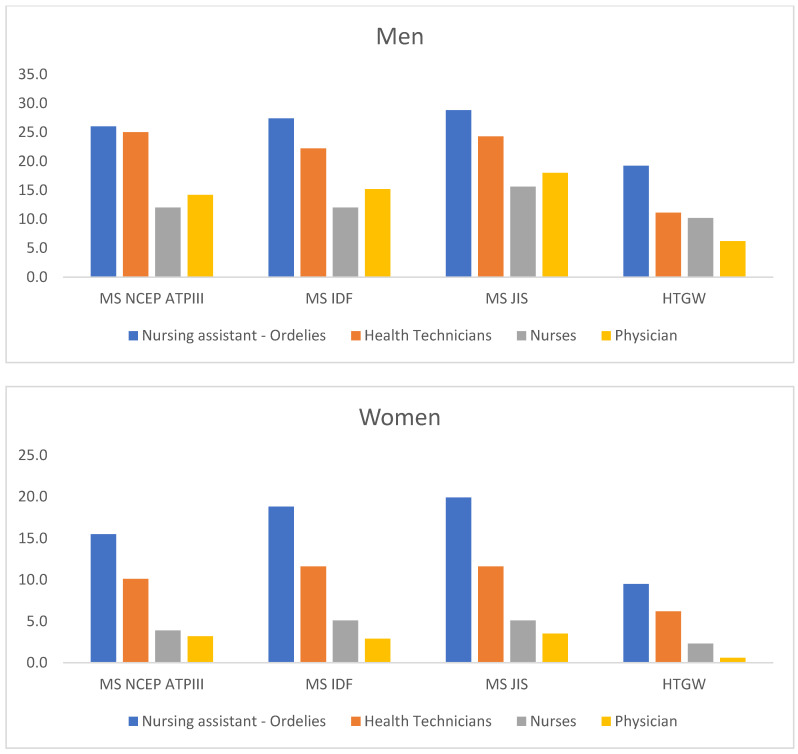
Prevalence of metabolic syndrome and hypertriglyceridemic waist circumference according to professional categories by sex.

**Figure 3:**
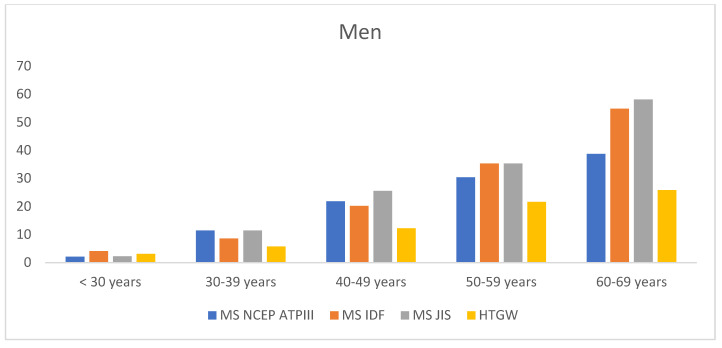

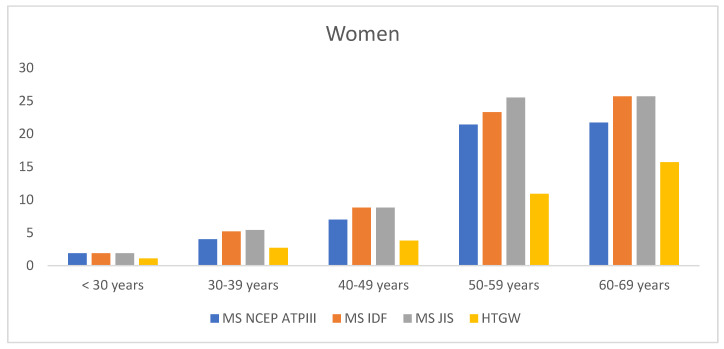
Prevalence of metabolic syndrome and hypertriglyceridemic waist circumference according to age by sex.

Table 2 present the prevalence of MetS (NCEP ATP III and JIS) according to different criteria and hypertriglyceridemic waist (HTW). An increase in prevalence is observed with advancing age and lower socioeconomic status (Figure 2 and Figure 3). These values are also higher among smokers, sedentary individuals, and those with low adherence to the Mediterranean diet. In all cases, values are lower in women. All differences identified were highly statistically significant (*p* < 0.001).

Table 3 presents the results of the binary logistic regression analysis. All independent variables included in the model (age, sex, professional category, smoking, physical activity, and adherence to the Mediterranean diet) were associated with the presence of MetS across the three diagnostic criteria and with HTW. Among these, age, sex, and physical activity showed the strongest associations, as indicated by their odds ratio values.

The agreement—measured using Cohen’s kappa coefficient—between MetS diagnosed using the three criteria is quite high, exceeding 0.8. In contrast, the agreement between MetS and HTW is approximately 0.6. The complete data are presented in Table 4.

Table 5 displays the results of the retrospective longitudinal study, showing the prevalence of MetS and HTW in the pre- (2010) and post- (2019) periods. Differences in prevalence between these periods for both MetS and HTW increased with age, with lower socioeconomic status, and were more pronounced among smokers, individuals who do not engage in regular physical activity, and those with low adherence to the Mediterranean diet. In all cases, the results were highly significant *p* < 0.001, and differences in prevalence were smaller in women.

## 4. Discussion

Our research found associations between MetS and HTW and all the sociodemographic variables and healthy habits analyzed. Identifying demographic, behavioral, and socioeconomic factors contributing to the risk of developing these conditions is crucial for designing preventive strategies. The scientific literature has explored how factors such as age, sex, socioeconomic status (SES), tobacco use, physical activity, and diet—particularly adherence to the Mediterranean dietary pattern—are related to MetS and HTW [38]. Studies conducted among healthcare workers, a population with unique work and health-related habits, provide a relevant context for understanding these associations [39].

The relationship between age and MetS, as observed in our study and previous research, is well documented, showing a progressive increase in prevalence with aging [40]. Global epidemiological studies using criteria such as NCEP-ATP III, IDF, and JIS consistently confirm that older adults are more likely to develop abdominal obesity, dyslipidemia, hypertension, and glucose abnormalities, the fundamental components of MetS. This phenomenon is closely associated with physiological changes linked to aging, such as the progressive decline in muscle mass (sarcopenia), increased visceral adiposity, impaired insulin sensitivity [41], and hormonal changes [42]. Furthermore, factors such as a sedentary lifestyle, hormonal imbalances, and heightened oxidative stress also contribute to this risk. These changes not only exacerbate metabolic complications but also increase the likelihood of cardiovascular diseases and type 2 diabetes [43].

Among healthcare workers, this trend is also evident, although with specific nuances. For instance, a study of middle-aged nurses revealed that MetS prevalence significantly increased after 40 years of age, aligning with data from the general population [44]. A study conducted in Nigeria compared the prevalence of MetS between healthcare and non-healthcare workers, finding similar prevalence rates in both populations. However, when analyzing the specific components of MetS, it was observed that abdominal obesity, elevated total cholesterol, and elevated LDL cholesterol were significantly more common among healthcare workers compared to non-healthcare workers [45]. These findings highlight potential differences in lifestyle or occupational factors that may predispose healthcare personnel to higher metabolic risks. Factors such as night shift work [46] and chronic stress unique to this group may accelerate the onset of metabolic disorders associated with aging [47].

Sex plays a crucial role in the presentation and prevalence of MetS and HTW in our study and others, with marked differences in susceptibility to their individual components [48]. Generally, men have a greater prevalence of MetS in early life, attributed to higher levels of visceral obesity and triglycerides [49]. Conversely, women experience a notable increase in prevalence post-menopause, likely linked to hormonal changes that promote abdominal fat deposition and insulin resistance [50]. Studies among healthcare workers have found that women, particularly nurses and healthcare assistants, are at a higher risk of central obesity and HTW compared to their male counterparts. This disparity may be attributed to work-related factors such as long working hours, the disruption of circadian rhythms, differences in dietary habits, and sleep duration and quality—an independent risk factor for MetS [51]. Additionally, limited time for physical activity further contributes to this increased risk [52].

The socioeconomic level is significantly associated with MetS and HTW risk in our study. Research indicates that individuals with lower SES have a higher prevalence of MetS, a trend consistently observed in studies applying NCEP-ATP III, IDF, and JIS criteria [53]. They indicate that MetS is more prevalent among individuals with lower socioeconomic status, influenced by factors such as age, gender, and education level. Older male workers and those in lower occupational classes are at a higher risk, particularly those with longer tenure in the same job [54,55].

This association is closely linked to higher rates of obesity, poorer diet quality, and limited access to health promotion programs. Previous studies have highlighted that socioeconomic factors play a crucial role in shaping dietary habits, significantly influencing individuals’ food choices. Specifically, factors such as educational attainment, possession of a university degree, occupation, and household income directly impact the consumption of healthy foods, particularly fruits and vegetables. These disparities underscore the importance of implementing strategies to address social inequities, improve diet quality, and reduce obesity risk [56].

In the case of healthcare workers, socioeconomic status plays a pivotal role in their metabolic health. While it might be assumed that this group, due to their training, has greater knowledge of healthy habits, studies reveal a different reality. Those in lower-paying positions, such as nursing assistants, exhibit a higher prevalence of MetS and HTW compared to higher-ranking professionals, such as physicians and nurses. This highlights how economic disparities within the healthcare sector also influence the ability to adopt healthier lifestyles, mirroring the broader inequalities observed in the general population [57]. Factors such as reduced job flexibility and financial stress may play a critical role [39,45].

Tobacco use was associated with increased MetS and HTW prevalence in our results. While some early studies suggested that smokers might have lower body weight than non-smokers [58], recent research demonstrates that smoking is linked to specific lipid abnormalities, such as elevated triglycerides and reduced HDL cholesterol—key components of MetS and HTW [59]. Smoking is significantly associated with MetS in young populations, doubling the risk compared to non-smokers. Smokers exhibit elevated triglyceride levels [60] and reductions in HDL cholesterol, factors that contribute to MetS. Additionally, smoking stimulates hormones such as cortisol and catecholamines, increases lipolysis, and elevates free fatty acid levels, which damage pancreatic beta cells and impair fasting glucose regulation [61]. Nicotine promotes fat breakdown, exacerbating these metabolic effects. Smokers face a higher risk of dyslipidemia than non-smokers, and former smokers also show an increased predisposition, indicating a lasting relationship between smoking and lipid alterations [62]. These results agree with those of our study in which we found a higher percentage of MetS and HTW in smokers. Among MetS components, abdominal obesity, hypertension, and impaired fasting glucose are more prevalent in smokers. These associations highlight the metabolic impact of current and past smoking, underscoring its role in the etiology of MetS. The analysis concludes that smoking is intrinsically linked to dyslipidemia and MetS development, regardless of smoking duration. This emphasizes the need for preventive interventions aimed at reducing smoking as a key strategy to mitigate the risk of MetS and its associated complications [63]. Additionally, smoking promotes a pro-inflammatory state and insulin resistance, contributing to cardiometabolic risk. One study found significantly higher HTW prevalence among smokers, particularly young men [11].

Regular physical activity emerges as one of the most protective factors against the development of MetS and HTW, as reflected in our findings. In our study, individuals who do not engage in regular exercise exhibit a significantly higher prevalence of MetS and HTW in both sexes. Binary logistic regression analyses confirm that physical inactivity is the most influential modifiable risk factor for the development of both MetS and HTW, with odds ratios (OR) ranging from 2.65 to 2.84. These results highlight the critical importance of promoting active lifestyles as a key strategy for preventing metabolic disorders, particularly in populations more vulnerable to sedentary behavior and its associated consequences. Evidence suggests that exercise improves insulin sensitivity, reduces visceral adiposity, and enhances lipid profiles by lowering triglycerides and increasing HDL cholesterol. It also benefits blood pressure and glucose control [64]. However, adherence to physical activity can be challenging for healthcare workers due to the demanding nature of their jobs. Studies in this population have reported insufficient physical activity levels and high sedentary behavior during leisure time, contributing to MetS and HTW, especially among women and night-shift workers [65,66].

Overnutrition and a diet high in fats and sugars are associated with metabolic syndrome (MetS) through the generation of ceramides and mitochondrial dysfunction. Interventions should include hypocaloric diets rich in monounsaturated fatty acids (MUFAs), polyunsaturated fatty acids (PUFAs), fruits, plant-based proteins, low-sugar yogurt, and medium-chain fats to promote optimal metabolic outcomes [67]. Among the dietary patterns that meet the requirements for preventing metabolic syndrome (MetS), the Mediterranean diet stands out as a widely recognized approach for its health benefits. This dietary pattern has been consistently associated with a significant reduction in the risk of MetS, according to the NCEP-ATP III, IDF, and JIS criteria, as well as notable improvements in components related to abdominal obesity (HTW). The Mediterranean diet is characterized by a high intake of fruits, vegetables, legumes, nuts, fish, and olive oil, along with the moderate consumption of wine, primarily during meals. This dietary approach not only promotes a healthier lipid profile but also enhances insulin sensitivity, reduces systemic inflammation, and aids in the prevention of cardiovascular diseases. Its abundance in antioxidants and healthy fats makes it a key tool for addressing MetS and other metabolic disorders [68]. In our study, we evaluated adherence to the Mediterranean diet as a protective factor against metabolic syndrome (MetS) and HTW. Our results reveal a strong association between low adherence to this diet and the presence of MetS and HTW, highlighting the importance of following this dietary pattern as a key preventive measure. In fact, insufficient adherence to the Mediterranean diet is, in our findings, the second most influential modifiable risk factor in the development of these conditions, following physical inactivity.

Among healthcare workers, adherence to the Mediterranean diet varies significantly depending on geographic region and workload. For example, studies conducted in Spain have shown that doctors and nurses who follow this dietary pattern have a lower prevalence of MetS and better metabolic markers compared to those who follow more common Western diets, such as those high in saturated fats and sugars. However, in other contexts, adherence to the Mediterranean diet remains limited, highlighting the urgent need to implement workplace interventions that promote healthy eating among healthcare workers [69]. A factor negatively influencing adherence to this healthy dietary pattern is shift work, as shift workers tend to have a higher percentage of unhealthy lifestyle habits, such as smoking, excessive alcohol consumption, physical inactivity, and unhealthy diets. All of these variables are closely related to an increased risk of MetS and HTW [27].

The interest in healthcare workers as a study population lies in their unique characteristics. On the one hand, this group is expected to have greater knowledge and awareness of chronic disease prevention. On the other hand, factors such as work stress, irregular shifts, and long hours may predispose them to unhealthy lifestyles. Studies often indicate that approximately 25% of healthcare workers meet MetS criteria, such as those defined by NCEP-ATP III. Differences in MetS prevalence between occupational roles—such as lower rates among physicians compared to nurses and aides—are often attributed to factors like education level, job stress, and access to health resources. For instance, research in Spain (e.g., the MESYAS registry) has identified significant MetS prevalence in workplace settings, especially among sedentary workers or those experiencing high levels of stress [70].

The significance of this study lies in the need to highlight and understand some of the non-communicable diseases (NCDs) affecting healthcare workers. Although often idealized due to their professional role, this group faces challenges similar to those of the general population, compounded by stressors inherent to their work environment. It is crucial to recognize that healthcare workers are individuals subjected to a unique combination of pressures, including long hours, night shifts, critical decision making regarding patient health, and frequent exposure to death and suffering. Additionally, they are often subject to constant scrutiny, which can contribute to significant physical and emotional exhaustion.

These factors do not operate independently but interact in complex ways to influence the risk of developing MetS and HTW. For instance, a healthcare worker on night shifts not only experiences circadian rhythm disruptions but may also develop unhealthy eating habits, opting for less nutritious foods due to a lack of suitable options during shifts. Similarly, the lack of regular physical activity and negative emotional states such as stress and anxiety further amplify this risk.

In this context, a crucial question arises: who cares for the caregiver? Ensuring the health of healthcare workers must be a priority at both the political and healthcare management levels. It is imperative to address the modifiable risk factors associated with NCDs in this group, such as smoking, harmful alcohol consumption, unhealthy diets, and physical inactivity. These factors, identified in our study, are key determinants in the development of MetS and HTW. Notably, physical inactivity emerged as the most influential variable. According to a study published in The Lancet in 2023, the global cost of physical inactivity is estimated to reach approximately 520 billion international dollars (INT$) over the decade 2020–2030 [71].

Promoting the health of healthcare workers not only directly benefits these professionals but also positively impacts the efficiency of the healthcare system and the quality of life of the general population. Preventing diseases in this group can lead to improved patient care, reduced healthcare costs, and a healthier society. Therefore, the dissemination and implementation of strategies to enhance the health of healthcare workers should be considered essential for ensuring the sustainability and quality of healthcare systems.

## 5. Strengths and Limitations

The strengths of this study include the large sample size (nearly 45,000 workers), making it one of the largest studies on healthcare workers worldwide. Additionally, it is one of the first, if not the first, to assess the prevalence of MetS (using three criteria) and HTW simultaneously across different healthcare roles. The variety of variables analyzed —sociodemographic and lifestyle-related—combined with the longitudinal design, allows for causal relationships to be established.

Another strength is having a reliable version of questionnaires to assess physical activity and adherence to the Mediterranean diet, providing a useful, cost-effective, and practical tool for evaluation and follow-up.

A limitation is the exclusion of unemployed individuals, retirees, and those under 18 or over 69 years old. While this exclusion limits generalizability to the broader population, we believe the large sample size mitigates this impact.

Likewise, since this is a population of Spain only, it is possible that the results may differ in other types of populations. Therefore, our results cannot be extrapolated to them.

Another limitation of this study is the use of self-administered questionnaires, as there are inherent limitations to self-reporting, such as recall bias and sensitivity to social desirability bias. Cross-validating this information with objective methods could be considered for future studies.

Other confounding factors such as comorbidities or pharmacological treatments were not included, as these data were not available.

## 6. Conclusions

The association between socioeconomic factors, lifestyle habits, and cardiometabolic risk (MetS and HTW) is particularly significant among healthcare workers, a vital group for public health. This population faces occupational risks such as long work hours, rotating shifts, constant stress, and limited access to health resources, all of which impact their metabolic health. Additionally, socioeconomic factors such as educational level, income, and occupation either exacerbate or alleviate this risk.

Implementing effective strategies for this group is a priority. Promoting a Mediterranean diet, increasing physical activity, and reducing tobacco and alcohol consumption are key interventions. Addressing occupational stress through mindfulness programs or flexible schedules could also improve their quality of life and overall health. These measures would not only benefit healthcare workers but also enhance the efficiency of healthcare systems, improving the quality of care delivered to the population.

Furthermore, it is essential for future studies to delve deeper into the causal relationship between lifestyle factors and the severity of MetS and HTW in healthcare workers. Understanding these dynamics would enable the development of more personalized and effective prevention and treatment plans tailored to the specific needs of this group. This approach would not only improve the health of healthcare workers but also set a positive example for the population they serve.

## Figures and Tables

**Table 1:** The characteristics of the population.

	Men, *n* = 14,305	Women, *n* = 30,634	
	**Mean (SD)**	**Mean (SD)**	***p*-Value**
Age (years)	41.1 (10.6)	40.4 (10.5)	<0.001
Height (cm)	176.0 (7.5)	162.6 (6.0)	<0.001
Weight (kg)	81.2 (14.5)	63.7 (13.3)	<0.001
Waist circumference (cm)	89.7 (12.6)	76.7 (11.8)	<0.001
Hip circumference (cm)	101.7 (8.8)	99.3 (10.7)	<0.001
Systolic blood pressure (mmHg)	128.2 (13.1)	116.1 (13.8)	<0.001
Diastolic blood pressure (mmHg)	79.9 (10.6)	74.8 (10.1)	<0.001
Total cholesterol (mg/dL)	191.8 (37.2)	187.8 (34.6)	<0.001
HDL-c (mg/dL)	48.9 (11.2)	59.3 (12.8)	<0.001
LDL-c (mg/dL)	165.2 (46.2)	144.8 (38.9)	<0.001
Triglycerides (mg/dL)	111.0 (73.2)	81.7 (47.0)	<0.001
Glucose (mg/dL)	93.6 (18.2)	88.9 (12.4)	<0.001
AST (U/L)	24.1 (17.2)	18.2 (8.0)	<0.001
ALT (U/L)	29.0 (36.7)	17.3 (13.7)	<0.001
GGT (U/L)	30.2 (28.8)	18.1 (18.1)	<0.001
	***N* (%)**	***N* (%)**	***p*-Value**
<30 years	2400 (16.8)	5984 (19.5)	<0.001
30–39 years	4200 (29.4)	8304 (27.1)	
40–49 years	4512 (31.5)	10,128 (33.0)	
50–59 years	2449 (17.1)	5150 (16.8)	
60–69 years	744 (5.2)	1120 (3.6)	
Physicians	5064 (35.4)	5024 (16.4)	<0.001
Nurses	4008 (28.0)	12,752 (41.6)	
Health technicians	1728 (12.1)	4128 (13.5)	
Nursing assistants and orderlies	3505 (24.5)	8782 (28.5)	
Non-smokers	12,001 (83.9)	26,094 (85.0)	<0.001
Smokers	2304 (16.1)	4592 (15.0)	
No physical activity	7512 (52.5)	18,744 (61.1)	<0.001
Presence of physical activity	6793 (47.5)	11,942 (38.9)	
Non-Mediterranean diet	7771 (54.2)	19,213 (62.1)	<0.001
Mediterranean diet	6534 (45.8)	11,413 (37.9)	

HDL—high-density lipoprotein. LDL—low-density lipoprotein. AST—Aspartate Aminotransferase. ALT—Alanine Aminotransferase. GGT—Gamma-glutamyl Transpeptidase. SD—standard deviation.

**Table 2:** Prevalence of metabolic syndrome and hypertriglyceridemic waist phenotype according to sociodemographic variables and healthy habits by sex.

		MS NCEP ATP III		MS IDF		MS JIS		HTW	
**Men**	***n***	**%**	***p*-Value**	**%**	***p*-Value**	**%**	***p*-Value**	**%**	***p*-Value**
<30 years	2400	2.1	<0.001	4.1	<0.001	2.2	<0.001	3.1	<0.001
30–39 years	4200	11.4		8.6		11.4		5.7	
40–49 years	4512	21.8		20.2		25.5		12.2	
50–59 years	2449	30.4		35.3		35.3		21.6	
60–69 years	744	38.7		54.8		58.1		25.8	
Physicians	5064	14.2	<0.001	15.2	<0.001	18.0	<0.001	6.2	<0.001
Nurses	4008	12.0		12.0		15.6		10.2	
Health technicians	1728	25.1		22.2		24.3		11.1	
Nursing assistants and orderlies	3505	26.0		27.4		28.8		19.2	
Non-smokers	12,001	17.2	<0.001	17.8	<0.001	20.2	<0.001	10.2	<0.001
Smokers	2304	20.8		19.8		24.0		15.6	
No physical activity	7512	25.2	<0.001	26.2	<0.001	29.1	<0.001	15.7	<0.001
Presence of physical activity	6793	9.5		9.2		11.7		6.0	
Non-Mediterranean diet	7771	23.6	<0.001	23.5	<0.001	27.2	<0.001	14.3	<0.001
Mediterranean diet	6534	11.0		11.3		13.6		6.8	
**Women**	***n***	**%**	***p*-Value**	**%**	***p*-Value**	**%**	***p*-Value**	**%**	***p*-Value**
<30 years	5984	1.9	<0.001	1.9	<0.001	1.9	<0.001	1.1	<0.001
30–39 years	8304	4.04		5.2		5.4		2.7	
40–49 years	10,128	7.0		8.8		8.8		3.8	
50–59 years	5150	21.4		23.3		25.5		10.9	
60–69 years	1120	21.7		25.7		25.7		15.7	
Physicians	5024	3.2	<0.001	2.9	<0.001	3.5	<0.001	0.6	<0.001
Nurses	12,752	3.9		5.1		5.1		2.3	
Health technicians	4128	10.1		11.6		11.6		6.2	
Nursing assistants and orderlies	8782	15.2		18.8		19.9		9.5	
Non-smokers	26,094	15.5	<0.001	8.6	<0.001	8.8	<0.001	3.9	<0.001
Smokers	4592	7.1		14.0		16.4		8.7	
No physical activity	18,744	10.3	<0.001	20.1	<0.001	12.6	<0.001	5.7	<0.001
Presence of physical activity	11,942	4.2		5.5		5.8		2.8	
Non-Mediterranean diet	19,213	9.5	<0.001	18.8	<0.001	11.5	<0.001	5.3	<0.001
Mediterranean diet	11,413	4.9		6.2		6.7		3.2	

MS—metabolic syndrome. NCEP ATP III—National Cholesterol Education Program Adult Treatment Panel III. IDF—International Diabetes Federation. JIS—Joint Interim Statement. HTW—hypertriglyceridemic waist phenotype.

**Table 3:** Binary logistic regression.

	MS NCEP ATP III	MS IDF	MS JIS	HTW
	OR (95% CI)	OR (95% CI)	OR (95% CI)	OR (95% CI)
Women	1	1	1	1
Men	3.31 (3.09–3.53)	2.82 (2.64–3.00)	3.17 (2.97–3.37)	3.72 (3.42–4.02)
<30 years	1	1	1	1
30–39 years	1.39 (1.23–1.55)	1.98 (1.76–2.20)	1.86 (1.66–2.07)	1.91 (1.66–2.16)
40–49 years	3.35 (2.96–3.75)	3.38 (3.08–3.68)	4.46 (3.99–4.93)	4.71 (4.08–5.34)
50–59 years	6.20 (5.42–6.99)	5.09 (4.53–5.66)	6.80 (6.11–7.50)	8.31 (7.07–9.55)
60–69 years	13.73 (11.23–16.23)	10.37 (9.11–11.63)	9.07 (8.00–10.14)	13.42 (10.83–16.01)
Physicians	1	1	1	1
Nurses	1.19 (1.11–1.28)	1.26 (1.15–1.37)	1.28 (1.17–1.39)	1.40 (1.29–1.51)
Health technicians	1.96 (1.80–2.13)	1.95 (1.80–2.11)	1.85 (1.71–1.99)	1.70 (1.54–1.86)
Nursing assistants and orderlies	2.66 (2.42–2.91)	3.13 (2.85–3.42)	2.71 (2.48–2.95)	5.15 (4.50–5.80)
Non-smokers	1	1	1	1
Smokers	1.27 (1.17–1.37)	1.18 (1.14–1.23)	1.32 (1.22–1.42)	1.55 (1.42–1.68)
Presence of physical activity	1	1	1	1
No physical activity	2.78 (2.58–2.99)	2.84 (2.65–3.04)	2.65 (2.48–2.83)	2.40 (2.19–2.62)
Mediterranean diet	1	1	1	1
Non-Mediterranean diet	2.01 (1.83–2.19)	2.05 (1.88–2.23)	2.10 (1.81–2.40)	1.91 (1.70–2.12)

MS—metabolic syndrome. NCEP ATP III—National Cholesterol Education Program Adult Treatment Panel III. IDF—International Diabetes Federation. JIS—Joint Interim Statement. HTW—hypertriglyceridemic waist phenotype. OR—odds ratio.

**Table 4:** Cohen’s kappa coefficient.

	MS NCEP ATP III	MS IDF	MS JIS	HTW
MS NCEP ATP III	1	0.831	0.891	0.597
MS IDF		1	0.949	0.638
MS JIS			1	0.595
HTW				1

MS—metabolic syndrome. NCEP ATP III—National Cholesterol Education Program Adult Treatment Panel III. IDF—International Diabetes Federation. JIS—Joint Interim Statement. HTW—hypertriglyceridemic waist phenotype.

**Table 5:** Differences in the prevalences of metabolic syndrome and hypertriglyceridemic waist between the pre- and post-periods by sex.

		MS NCEP ATP III			MS IDF			MS JIS			HTGW		
**Men**	***n***	**%Pre–Post**	**Difference %**	***p*-Value**	**%Pre–Post**	**Difference %**	***p*-Value**	**%Pre–Post**	**Difference %**	***p*-Value**	**%Pre–Post**	**Difference %**	***p*-Value**
<30 years	2400	2.0–2.1	3.5	<0.001	3.9–4.1	3.9	<0.001	2.1–2.2	4.7	<0.001	2.9–3.1	4.9	<0.001
30–39 years	4200	10.8–11.4	4,9		8.2–8.6	5.2		10.7–11.4	5.9		5.4–5.7	6.1	
40–49 years	4512	19.9–21.8	8,9		18.3–20.2	9.3		22.9–25.5	10.2		10.9–12.2	10.8	
50–59 years	2449	27.0–30.4	11,3		31.1–35.3	11.8		30.6–35.3	13.2		18.5–21.6	14.2	
60–69 years	744	32.2–38.7	16,8		45.3–54.8	17.3		46.8–58.1	19.5		20.6–25.8	20.3	
Physicians	5064	13.7–14.2	3,8	<0.001	14.6–15.2	4.1	<0.001	17.0–18.0	5.8	<0.001	5.8–6.2	5.8	<0.001
Nurses	4008	11.4–12.0	4,6		11.4–12.0	4.7		14.6–15.6	6.2		9.6–10.2	6.2	
Health technicians	1728	23.1–25.1	7,9		20.4–22.2	8.2		21.7–24.3	10.8		9.7–11.1	12.5	
Nursing assistants and orderlies	3505	23.4–26.0	10,1		24.5–27.4	11.5		24.2–28.8	15.9		16.0–19.2	16.7	
Non-smokers	12,001	16.0–17.2	6,8	<0.001	16.6–17.8	7.0	<0.001	18.6–20.2	8.1	<0.001	9.4–10.2	7.9	<0.001
Smokers	2304	18.9–20.8	8,9		17.0–19.8	9.1		21.5–24.0	10.3		14.0–15.6	10.3	
No physical activity	7512	21.2–25.2	15,8	<0.001	21.9–26.2	16.3	<0.001	23.9–29.1	17.8	<0.001	12.7–15.7	19.1	<0.001
Presence of physical activity	6793	9.2–9.5	3,2		8.8–9.2	4.4		11.1–11.7	5.1		5.7–6.0	5.3	
Non-Mediterranean diet	7771	20.2–23.6	14,4	<0.001	20.0–23.5	15.1	<0.001	22.7–27.2	16.7	<0.001	11.8–14.3	17.8	<0.001
Mediterranean diet	6534	10.5–11.0	4,6		10.7–11.3	5.1		12.8–13.6	5.9		6.4–6.8	6.2	
**Women**	***n***	**%**		***p*-Value**	**%**		***p*-Value**	**%**		***p*-Value**	**%**		***p*-Value**
<30 years	5984	1.8–1.9	1.9	<0.001	1.9–1.9	2.1	<0.001	1.8–1.9	2.0	<0.001	1.1–1.1	2.4	<0.001
30–39 years	8304	3.9–4.0	2.8		5.0–5.2	2.9		5.2–5.4	3.1		12.3–2.7	3.2	
40–49 years	10,128	6.6–7.0	5.9		8.3–8.8	6.0		8.3–8.8	5.8		3.6–3.8	6.2	
50–59 years	5150	19.7–21.4	7.9		21.4–23.3	8.2		23.3–25.5	8.5		9.9–10.9	8.8	
60–69 years	1120	19.1–21.7	11.2		22.9–25.7	10.9		22.9–25.7	11.0		13.8–15.7	12.0	
Physicians	5024	3.1–3.2	3.1	<0.001	2.8–2.9	3.0	<0.001	3.4–3.5	3.2	<0.001	0.6–0.6	3.4	<0.001
Nurses	12,752	3.6–3.9	4.0		4.9–5.1	4.1		4.8–5.1	5.6		2.2–2.3	5.0	
Health technicians	4128	9.1–10.1	6.7		10.8–11.6	7.0		10.8–11.6	7.2		5.7–6.2	7.4	
Nursing assistants and orderlies	8782	14.0–15.5	9.9		17.9–18.8	9.8		18.0–19.9	9.6		8.6–9.5	9.7	
Non-smokers	26,094	14.8–15.5	4.4	<0.001	8.2–8.6	4.3	<0.001	8.4–8.8	4.6	<0.001	3.7–3.9	4.1	<0.001
Smokers	4592	6.6–7.1	6.6		13.1–14.0	6.5		15.3–16.4	6.9		8.1–8.7	6.8	
No physical activity	18,744	9.1–10.3	11.6	<0.001	17.9–20.1	10.9	<0.001	11.2–12.6	11.2	<0.001	5.1–5.7	10.9	<0.001
Presence of physical activity	11,942	4.1–4.2	3.2		5.3–5.5	3.2		5.6–5.8	3.5		2.7–2.8	3.1	
Non-Mediterranean diet	19,213	8.5–9.5	11.0	<0.001	16.8–18.8	10.4	<0.001	10.3–11.5	10.4	<0.001	4.8–5.3	10.1	<0.001
Mediterranean diet	11,413	4.7–4.9	3.4		6.0–6.2	3.7		6.5–6.7	3.6		3.1–3.2	3.7	

MS—metabolic syndrome. NCEP ATP III—National Cholesterol Education Program Adult Treatment Panel III. IDF—International Diabetes Federation. JIS—Joint Interim Statement. CHTGW—hypertriglyceridemic waist phenotype.

## Data Availability

This study’s data are stored in a database that complies with all security measures at ADEMA-Escuela Universitaria. The Data Protection Delegate is Ángel Arturo López González.
